# West Nile Virus and Usutu Virus Co-Circulation in Europe: Epidemiology and Implications

**DOI:** 10.3390/microorganisms7070184

**Published:** 2019-06-26

**Authors:** Silvia Zannoli, Vittorio Sambri

**Affiliations:** 1 Unit of Microbiology, The Great Romagna Area Hub Laboratory, Pievesestina, 47522 Cesena, Italy; 2 Department of Experimental, Diagnostic and Specialty Medicine (DIMES), University of Bologna, 40138 Bologna, Italy

**Keywords:** West Nile, Usutu, epidemiology, co-circulation

## Abstract

West Nile virus (WNV) and Usutu virus (USUV) are neurotropic mosquito-borne flaviviruses that may infect humans. Although WNV is much more widespread and plays a much larger role in human health, the two viruses are characterized by similar envelope antigens, clinical manifestations, and present overlapping in terms of geographic range of transmission, host, and vector species. This review highlights some of the most relevant aspects of WNV and USUV human infections in Europe, and the possible implications of their co-circulation.

## 1. Introduction

West Nile virus (WNV) and Usutu virus (USUV) are neurotropic mosquito-borne flaviviruses within the family *Flaviviridae*. The genus *Flavivirus* is comprised of more than 70 recognized viruses, including some of the most significant arboviral pathogens of humans. Both are members of the Japanese encephalitis virus (JEV) serocomplex, sharing cross-neutralization antibodies with other important viruses that cause encephalitis in humans, as well as viruses that are either rare or less well-established causes of disease [1].

WNV and USUV are maintained by a sylvatic cycle between different species of birds as amplifying hosts and *Culex* mosquitoes as major vectors. Humans and other mammals may be infected by mosquitoes; however, they are not able to sustain transmission and are therefore considered dead-end hosts. WNV infection represents nonetheless a serious burden to human and animal health because of its capacity to cause unforeseen and large epidemics [2].

In their transmission cycle, the two viruses share some of the same vectors and hosts, and co-circulation in the same environment has been reported [3]. USUV appears to be more pathogenic and lethal for some bird species compared with WNV, while it rarely causes disease in humans. However, the clinical manifestations show many similarities, which may complicate the diagnosis of febrile conditions. This review highlights some of the most relevant aspects of WNV and USUV human infections in Europe, and the possible implications of their co-circulation.

## 2. Transmission

The natural life cycle of WNV and USUV is similar to that of other flaviviruses belonging to the JEV serocomplex [4], as it involves ornithophilic mosquitoes as vectors and birds as main amplifying hosts and, under certain environmental conditions, it spills over to human settlements. Mammals, including humans and equines, can incidentally become infected with both viruses, but they generally do not develop sufficient viraemia to sustain transmission [5,6].

The main vector in outbreaks of WNV infection in Europe seems to be *Culex pipiens* [7]. USUV co-circulates with WNV in many European countries, in terms of geographic range of transmission, host and vector species, since *Cx. pipiens* is also the most common vector for USUV [8].

The overlap in the range of bird hosts is substantial: 34 species of 11 orders have been shown to be susceptible to both WNV and USUV [3]. Most of the species found susceptible to infection naturally occur in Europe, such as Eurasian jays (*Garrulus glandarius*), common starlings (*Sturnus vulgaris*), and Eurasian magpies (*Pica pica*) [3]. Half of the identified species are at least partially migratory, which plays a part in the circulation and diffusion of both viruses.

In the great majority of cases, WNV infection is acquired as a consequence of the bite of a mosquito. Alternative modes of transmission have been reported, such as organ transplants, blood transfusions, and vertical transmission either during pregnancy, delivery, or breastfeeding [9]. These routes of transmission have not been described for USUV yet.

## 3. Virology

### 3.1. WNV

WNV is an enveloped virus with icosahedral symmetry [10]. Image reconstructions and electron microscopy showed its structure is similar to the dengue fever virus, characterized by a 45–50 nm virion [10]. The RNA genome is linear, plus sense, single-stranded, and approximately 11 kb long. It is flanked by 5′ and 3′ non-coding stem loop structures, with a clustering of coding regions for structural proteins at the 5′ end and nonstructural proteins at the 3′ end. The envelope is characterized by glycoprotein E and pre-membrane protein (prM). The former mediates binding to the host cells and promotes viral entry into the host cells, while the latter is necessary for virion assembly and maturation by assisting envelope folding [10].

The nucleocapsid is composed of C proteins, each 105 aa in size, which form the capsid and are bound to the genomic RNA.

Replication takes place in the cytoplasm of the host cell. Here, the nucleic acid of the virus is translated into a single polyprotein; after this step, cellular and viral enzymes proceed to the cleavage of the polyprotein into both functional and structural proteins.

WNV is part of the JEV serocomplex, sharing cross-neutralization antibodies with viruses including Japanese encephalitis virus, Murray Valley encephalitis virus, and St Louis encephalitis virus, which are able to cause encephalitis in humans, as well as viruses which are rarely cause of human disease, such as Usutu [1].

### 3.2. USUV

Like WNV and most other flaviviruses, USUV virions are small and spherical, with a diameter of 40–60 nm, consisting of a dense core with an adherent lipid envelope derived from host cell membrane. USUV is a positive-sense, single-stranded RNA virus. The genome is about 11 kb in length, has no 3′ poly(A) tail, and shows a similar organization to others flaviviruses [11], with a 5′ cap structure, a unique open reading frame (ORF), and two untranslated regions (UTRs), which may vary in length among different strains (95–96 nt for 5′, and 631–664 nt for 3′) [12], and are involved in the translation and replication processes. The ORF is translated in a single polyprotein which is post-translationally processed into eleven proteins, of which three are structural (capsid, envelope, and pre-membrane) and eight nonstructural. Like other flaviviruses, the genes encoding for structural proteins are located on the 5′ end of viral genome [4,13]. The capsid proteins (C) form the central core of the virion and are associated with the viral RNA.

The nonstructural proteins cater to different functions during infection, which were deduced on the basis of their similarity with other flavivirus genomes [14]. NS1 exists in both a cellular and a secreted form, and is necessary for replication and virion maturation [15]. NS2A, NS2B, NS4A, and NS4B play a role in the assembly of the virus and the inhibition of interferon response [16]. NS3 and NS5 present enzymatic activity.

## 4. Clinical Aspects

### 4.1. WNV

WNV-associated illness was initially considered to be a negligible disease, even asymptomatic; however, it became a public health issue at the end of the 1990s as severe, even fatal, neurological manifestations followed several outbreaks of infection [17,18]. Understanding of the clinical epidemiology of WNV infection is complicated by the high number of asymptomatic cases, as well as patients that fail to seek medical attention due to symptoms being perceived as mild. However, it is estimated that around 30% of infected people develop West Nile fever, with symptoms ranging from a flu-like syndrome to encephalitic diseases, and reported fatality rates range from 3% to 17% [19,20]. The symptomatology has been better defined by a 2010 study as the presence of at least three indicator symptoms, which involve generalized weakness, severe muscle and joint pain, fever, headache, painful eyes, or new rash [21].

Neuroinvasive disease is a potentially fatal complication of epidemic WNV infection, presenting as encephalitis, meningitis, or acute flaccid paralysis. This is developed by less than 1% of the infected individuals, although the proportion increases with age [22,23]. Some population groups were reported to be at greater risk of severe neurological disease or death: along with the elderly, male patients with underlying diseases, such as cardiovascular conditions, cancer, or diabetes, were shown to be at increased risk of neuroinvasive disease compared with female patients without underlying disease [24]. Moreover, the genetic variation of WNV is important in determining its pathogenicity, including the tendency to invade the meninges [25]. The fatality rate in neuroinvasive cases is approximately 10% [26].

Present evidence, gathered on the basis of nucleic acid homology, supports the thesis that WNV divides into at least seven lineages, where the major divergence in nucleotide sequence is of 25%–30%. Lineages 1 and 2 present a homology in nucleotide sequence of around 75%; these are the only lineages so far associated with human disease [24].

Unlike acute morbidity and mortality, the long-term sequelae associated with WNV infection were not well characterized until 2015, when the clinical outlook of WNV-related illness in North America and Western Europe was reviewed [27]. The evidence suggested that WNV-related illness is associated with severe long-term outcomes. Although the incidence, length, and nature of the sequelae were shown to be highly variable, patients with WNV-related neuroinvasive manifestations or with West Nile fever presented persistent signs of cognitive, functional, and physical nature [27].

The most common physical sequelae consisted of muscle weakness, fatigue, and myalgia; memory loss, depression, and difficulty concentrating were among the most common cognitive long-term effects, while difficulties ambulating or with daily activities were the most reported functional sequelae (Table 1). Lengthy recovery seemed to be common [27].

At present, there are no available human vaccines or specific antiviral treatments for WNV-related disease. Severely affected patients may need to be hospitalized to receive supportive treatment, while over-the-counter pain relievers are used in milder cases. Community-level mosquito control programs to reduce vector densities and repellents are the most efficient precautionary measures. Most European countries have implemented passive or active surveillance networks which have improved the quality of available epidemiological data. Nonetheless, outbreaks remain temporally and spatially unpredictable [28].

### 4.2. USUV

Although far less common, the clinical manifestations of USUV infection are rather similar to WNV. Infection in humans may be asymptomatic or be associated to a wide range of symptoms which vary from moderate (rash, fever, and headache) to severe, presenting as meningoencephalitis, encephalitis, polyneuritis, or facial paralysis [29,30,31].

USUV can be divided into eight lineages which are distinct into an African and a European group based on their origin of isolation [12]. The nucleotide identity between isolates is higher than 94%, with the exception of the lineage Africa 1, which only includes the strain CAR-1969, with an identity of 78.3% [11,13].

As USUV and WNV show considerable similarities and that an accurate laboratory diagnosis presents some difficulties, an underestimation of the burden of USUV-related disease might be likely.

## 5. Diagnosis

### 5.1. WNV

The clinical presentation of WNV infection is highly diverse and overlapping with other flaviviruses. Laboratory diagnosis is essential, with several techniques currently available to this end [32].

Molecular methods, usually real-time RT-PCR assays, are the most common diagnostic tool that is used to detect viral RNA in a variety of specimens. Identification of WNV genome in the cerebrospinal fluid (CSF) or serum of a patient during the acute stages of neurological manifestations is generally considered to be a confirmatory diagnostic parameter [33].

The laboratory diagnosis of WNV infection in humans still heavily relies on the detection of specific antibodies. When the presence of viral RNA is not demonstrable by molecular assays, the observation of a specific IgM immune response might be considered anindicator of the early stages of an infection. CSF or serum are the specimens of choice. The presence of IgM antibodies for WNV, however, is not necessarily a sign of an acute WNV infection as they have been reported to persist for more than a year in certain patients. A combination of IgM levels with IgG avidity determination has been shown to help differentiate a current WNV infection from IgM seropositivity due to the previous WNV transmission season [34].

Seroconversion during the convalescent phase of the infection (indicated by a four-fold or higher increase in the level of IgG in the serum) is required as confirmation. Immunofluorescence assays or enzyme immunoassays can be used to detect WNV-specific antibodies [32]. However, the widespread immunological cross-reactions among closely related flaviviruses impact unfavorably on these techniques, significantly lowering their specificity [35]. To overcome this limit, positive results should be confirmed with a plaque reduction neutralization assay (PRNT); only a few laboratories in Europe are capable of routinely performing this test, however, due to the complexity of the technique and the need for viable virus isolates, a BSL-3 safety condition is required [36].

Virus isolation in cell cultures from CSF and serum samples is a third potential diagnostic alternative. Although very specific, this technique is characterized by a low sensitivity and requires the continuous availability of cell cultures under BSL-3 conditions [37]. For these reasons, this method is generally only performed in research laboratories.

### 5.2. USUV

The same diagnostic issues presented for WNV infection are valid for USUV. USUV infection can be diagnosed directly, through the detection of viral nucleic acid or by virus isolation in cell culture, or indirectly, by targeting specific antibodies.

The direct diagnosis of acute USUV infection is based on the detection of USUV RNA in clinical specimens (blood and CSF) by nucleic acid amplification methods. Currently, there are no validated commercial assays available, although several in-house methods have been described, usually real-time RT-PCR assays which specifically target USUV [38,39]. Alternatively, a nested RT-PCR has been described which targets highly conserved genome sequences among flaviviruses [40], followed by the sequencing of amplicons to identify the virus.

Indirect diagnosis of USUV infection relies on serology. Antibody detection is performed through enzyme-linked immunoassays (ELISA) or immunofluorescence tests [41]; as for WNV, any positive result must be confirmed by more specific tests, such as PRNT, to rule out cross-reactivity with antibodies against closely related flaviviruses. Seroconversion should be demonstrated to confirm the infection.

## 6. Epidemiology

### 6.1. WNV

This virus was first isolated from the blood of a febrile patient in 1937 in the West Nile district of Uganda [42] and has subsequently spread worldwide. WNV has been circulating in Europe and in the Mediterranean Basin since at least the late 1950s [43], where it has caused both sporadic infections and outbreaks in humans [44]. Most of human and/or equine infections were characterized by moderate pathogenicity, with the exception of a large outbreak in 1996, reported in Romania with over 390 confirmed cases. This is the largest outbreak of WNV infection in humans to date inside the EU [45].

WNV lineage 1 was the only one involved in such manifestations until 2004, and the predominant lineage causing outbreaks up to 2010 [45,46,47]. WNV lineage 2 was initially isolated in 2004 (Hungary) and in 2008 (Austria), and then dispersed to other countries in central and southern Europe, gradually replacing WNV lineage 1 strains [48,49]. Analysis of WNV genome sequences demonstrated that WNV strains that previously caused outbreaks appeared to be displaced by new strains. This can be explained by positive selection, or by suitable ecological conditions that favored a particular viral strain [50].

Since 2010, WNV epidemiological pattern has evolved from a very low level of endemicity to a sudden increase of the incidence of animal and human neurological cases. A major WNF epidemic occurred in 2010 in Central Macedonia, Greece, with 262 clinical human cases and 35 fatalities [51].

The burden of WNV-related disease in Europe is relevant. According to epidemiological data from ECDC, a total of 1832 human cases of West Nile neuroinvasive disease were detected in the EU in the 2011–2017 period and in neighboring countries [52]. In 2018, 1503 human cases were reported in the EU/EEA and 580 cases by the EU neighboring countries, more than seven times the number of infections reported in 2017, with a proportion of West Nile neuroinvasive disease among symptomatic cases of 68% [53]. A total of 180 deaths have been reported. The total number of reported autochthonous infections exceeds the total number from the previous seven years [53]. Of interest is the fact that in almost all countries, the first cases were observed in areas close to wetlands, such as the Po river delta in Italy or the Axios river delta in Greece. Such areas attract migrating birds, as well as abundant *Culex* mosquito populations [26].

### 6.2. USUV

First isolated in South Africa in 1959 [54], USUV emerged for the first time in Europe in 1996 causing deaths among common blackbirds (*Turdus merula*) in the Tuscany region of Italy [55]. The first case of human USUV infection was reported in Central African Republic in 1981 [56].

A total of 25 human cases of USUV infection have been described to date (Table 2), with 22 cases of USUV-related neuroinvasive infection documented in Europe. Italy reported the highest number of cases (*n* = 15), but USUV infections were also found in Croatia, Germany, and France [57,58,59,60,61].

Recently, USUV infection has been described in healthy blood donors testing positive for WNV by nucleic acid amplification technique (NAT) in Germany, Italy, and Austria [60,62,63,64]. Most notably, Austria also reported for the first time a blood donor who was found to have a double infection with both WNV and USUV [64].

Seroprevalence studies on the subject have also provided evidence that the prevalence of USUV infection seems to be even higher than WNV infection in areas where both viruses co-circulate (7% and 3% respectively) [65,66].

## 7. Vectors

At present, four European mosquito species have been demonstrated as competent vectors for WNV (*Cx. pipiens*, *Cx. modestus*, *Aedes albopictus*, and *Aedes detritus*) [67,68,69,70]. *Cx. pipiens*, which is highly abundant during summer, showed the highest transmission rates [71], and is therefore considered the main vector for WNV in Europe with *Culex modestus* playing a role in specific regions [72]. The life cycles of the mosquito, virus, amplifying and incidental hosts, and the interactions between them, are influenced by environmental parameters, particularly temperature [73]. As a result of this, outbreaks of WNV infection are sporadic and focalized, showing high variability in their development and incidence [74].

Currently, mosquitoes belonging to 7 genera (*Aedes*, *Anopheles*, *Coquillettidia*, *Culex*, *Culiseta*, *Mansonia*, and *Ochlerotatus*) have been shown to be positive for USUV [3]. Like WNV, USUV is mainly transmitted by *Culex* mosquitoes and, among European mosquito species, it is mostly found in *Cx. pipiens* [8].

USUV and WNV co-circulate in parts of southern Europe, but the distribution of USUV extends into central and northwestern Europe. As human cases are virtually absent in northern Europe, bird and mosquito surveillance programs are necessary to monitor WNV in these regions. However, not all European countries implement routine surveillance plans, and most WNV infections in humans are asymptomatic, making WNV circulation likely to be underestimated [75].

The lower number of human WNV cases reported in northern Europe may be explained by a lower susceptibility of bird populations, or by a lower vectorial capacity of the mosquitoes. Data on the viraemia of birds obtained both in field and laboratory studies indicate that bird hosts do not seem to be a limiting factor for WNV transmission, so a lowered vectorial capacity in the mosquitoes might be an alternative explanation [75].

Vectorial capacity depends on several parameters, such as the feeding behavior, abundance, and survival of the mosquitoes, as well as the environment [76]. Vector competence also plays an important role in vectorial capacity: analysis of this aspect helps in identifying species that might be important contributors to WNV transmission and implementing control measures to reduce the potential of WNV transmission [77]. The vector competence of the mosquito to transmit a certain arbovirus is determined by a combination of vector species, and lineage and strain of the virus. For a mosquito exposed the virus to become infectious, WNV has to overcome a range of anatomical barriers [75,77] which can limit the infection either mechanically or through immune response.

While no intrinsic differences in vector competence are detectable between northern and southern European *Cx. pipiens* [78], temperature impacts strongly on vectorial capacity [77], and correlates positively with the dispersal of WNV [79]. In the temperature range from 18 to 28 °C, transmission rates of northern European *Cx. pipiens* have been shown to increase of approximately 30% [8].

Additionally, the species *Cx. pipiens* comprises two different biotypes, *pipiens* and *molestus*. The former shows a preference for avian hosts, while the *molestus* biotype is more likely to target mammals [80]. Therefore, the two biotypes are respectively important for the natural transmission cycle and in the spillover of WNV to humans. While no disparity has been shown in vector competence among the two biotypes, the response to temperature was different [78], which implicates an additional level of significance of this parameter, and therefore geography, in the dissemination of WNV.

Despite differences in infectivity and transmissibility, northwestern European mosquitoes show infection rates for USUV which are comparable with values obtained for WNV, with an overlapping distribution throughout Europe [8]. USUV activity is also found in more temperate regions, however, the infectivity in *Cx. pipiens* showed a stronger correlation to temperature compared to WNV. Additionally, at higher temperatures, mosquitoes were shown to be more effectively infected and therefore more competent for USUV (90% at 28 °C) than for WNV (58% at 28 °C [8].

## 8. WNV and USUV Co-Circulation

WNV is a well-known emerging pathogen and is considered the most widespread flavivirus globally [81]. USUV is less prominent, but still caused significant mortality among bird populations and has been detected in many European countries [4,82]. While the two viruses differ substantially in their significance for human health, they overlap on several aspects and may interact at the population level.

**Figure 1:**
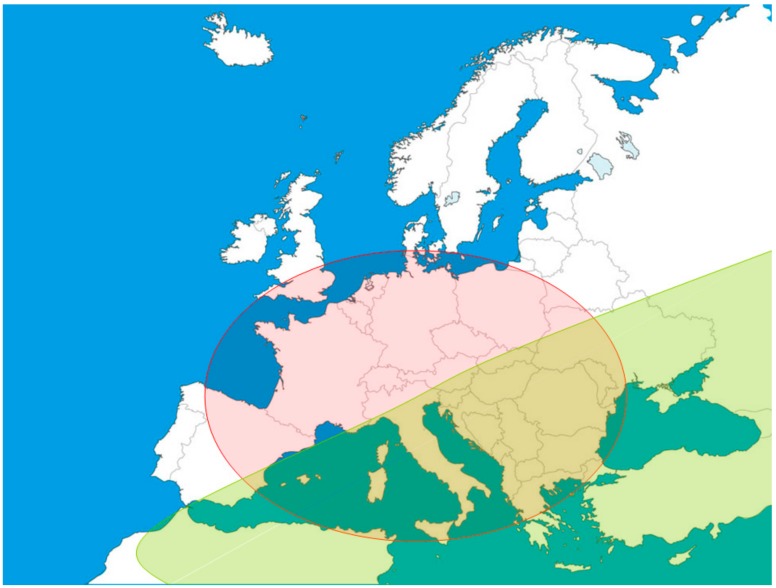
Geographic distribution of WNV (green) and USUV (red) in Europe. Co-circulation, paired with antigenic similarity and overlapping clinical expression of the infections, complicates the diagnosis of febrile conditions, leading to suboptimal case management. More importantly, this may directly alter the epidemiology of these viruses. This was shown in California, after the introduction of WNV caused the displacement of Saint Louis encephalitis virus (SLEV), and in Thailand, with an increased severity of dengue virus (DENV) infection symptoms in individuals with previous JEV infection [86,87].

Since the first report of USUV in Europe, the virus has spread to several European countries, overlapping substantially with the circulation of WNV (Figure 1). Co-circulation of WNV and USUV occurs in terms of species of vectors and amplifying hosts, as well as in the geographic range of distribution [81]. This implies the potential of WNV to spread to areas where only USUV has been observed to date, and viceversa [3], especially when paired with the fact that WNV and USUV were shown to be able to infect several bird species which are at least partially migratory [81].

Additionally, WNV and USUV envelope proteins are highly similar in terms of amino acid sequence [83,84]. This is the main target of the antibody responses to flaviviruses and implies a close antigenic relationship, which has been confirmed by seroneutralization as well as cross-reactivity and potential cross-immunity [85]. As previously demonstrated, subsequent infections by two closely related viruses, such as those belonging to the JEV serocomplex, can modify the susceptibility to infection and progression of disease [3].

Interestingly, five cases were reported of WNV infection in patients which presented USUV neutralizing antibodies at the time of the first evaluation [88]. These patients were characterized by an atypical immune response, with the presence of WNV RNA in blood and WNV IgG antibodies at the time of diagnosis, but a blunted or absent IgM antibody response during follow-up. A similar pattern of immune response was reported both in patients infected with DENV following a previous infection with a heterologous DENV serotype [89] and in patients with previous DENV immunity who were infected with Zika virus [90]. The reported cases were asymptomatic or presented mild fever, suggesting a lack of disease enhancement [88].

The current situation predicts more frequent and prolonged temperature anomalies. This aspect, paired with the presence of intrinsically competent vectors and susceptible avian hosts, prospects a context with no obvious restrictions which could impede WNV circulation in northern Europe [75]. Considering USUV and WNV share a main vector, *Cx. pipiens*, and present comparable transmission cycles and transmission rates, the circulation of USUV might be a prelude to WNV transmission [8].

Should the circulation of USUV have an effect on the transmission of WNV, a noteworthy human pathogen, this would present significant implications for public health. Whether these interactions can influence the patterns of virus circulation in Europe is yet to be clarified.

## Figures and Tables

**Table 1:** Most widely reported West Nile virus (WNV)-related sequelae (>5% patients).

**Physical Sequelae**	
15%–20% patients	Muscle weakness, fatigue, myalgia
10%–15% patients	Headache
5%–10% patients	Balance problems, visual impairment, joint weakness or pain, tremor, neck pain or stiffness
**Cognitive and Psychological Sequelae**	
15%–20% patients	Memory loss
10%–15% patients	Depression, difficulty concentrating
5%–10% patients	Agitation or increased sensitivity, confusion, altered mental status, aggressivity or anger, anxiety, emotional lability
**Functional Sequelae**	
40%–50% patients	Difficulty performing daily living activities
<10% patients	Decreased activity, difficulty ambulating

**Table 2:** Human cases of Usutu virus (USUV) infection.

Country	Year	Clinical Presentation	N° of Cases
Central African Republic	1981	Fever and rash	1
Burkina Faso	2004	Fever	1
Italy	2008–2009	Neuroinvasive disease	15
Croatia	2013–2018	Neuroinvasive disease	6
Germany	2016	None	1
France	2016	Neuroinvasive disease	1
